# Inflammatory markers in outpatients with schizophrenia diagnosis in regular use of clozapine: a cross-sectional study

**DOI:** 10.3389/fpsyt.2023.1269322

**Published:** 2023-10-09

**Authors:** Victor Hugo Schaly Cordova, Amelia Dias Teixeira, Ana Paula Anzolin, Roberta Moschetta, Paulo Silva Belmonte-de-Abreu

**Affiliations:** ^1^Faculty of Medicine, Graduate Program in Psychiatry and Behavior Science, Federal University of Rio Grande do Sul, Porto Alegre, Brazil; ^2^Clinical Hospital of Porto Alegre, Federal University of Rio Grande do Sul, Porto Alegre, Brazil; ^3^Institute of Basic Health Science, Graduate Program in Biological Science: Biohemestry, Federal University of Rio Grande do Sul, Porto Alegre, Brazil; ^4^Laboratory of Molecular Psychiatry, Clincal Hospital of Porto Alegre (HCPA), Porto Alegre, Brazil; ^5^Faculty of Medicine Undergraduate Course in Medine, Federal University of Rio Grande do Sul, Porto Alegre, Brazil; ^6^Psychiatry Service, Clinical Hospital of Porto Alegre, Federal University of Rio Grande do Sul, Porto Alegre, Brazil

**Keywords:** inflammatory response, neutrophil-linphocyte ratio, inflammatory markers, immunoinflammatory systemic index, psychopharmacology, second generation antipsychotic

## Abstract

It is known that inflammation worsen the course of schizophrenia and induce high clozapine serum levels. However, no study evaluated this change in function of clozapine daily dose in schizophrenia. We assessed the correlation between inflammation and severity symptoms in patients with schizophrenia that take and do not take clozapine. We also assessed the correlation between clozapine daily dose and inflammatory markers to patients who take this drug. Patients were recruited from Schizophrenia Ambulatory and Psychosocial Care Center of Clinical Hospital of Porto Alegre and from an association of relatives of patients with schizophrenia. Exam results, and other important clinical exam were assessed in patients record or patients were asked to show their exam in the case of outpatients. We included 104 patients, 90 clozapine users and 14 non-clozapine users. We calculate the systemic inflammatory markers [neutrophil-lymphocyte ratio (NLR), systemic immune inflammation index (SII), and the psychopathology severity by the Brief Psychiatric Rating Scaled anchored (BPRS-a)]. These variables were compared between clozapine users and non-clozapine users. It was used mean/median test according to data distributing, with study factor (SII, MLR, and PLR), the clinical outcome: severity of symptomatology (BPRS score), and clozapine daily dose as adjustment factor. Clozapine users exhibited a significantly higher neutrophil count (mean ± SD: 5.03 ± 2.07) compared to non-clozapine users (mean ± SD: 3.48 ± 1.27; *p* = 0.031). After controlling for comorbidity, other parameters also showed significant differences. These findings are consistent with previous studies that have demonstrated an inflammatory response following the administration of clozapine.

## Introduction

Schizophrenia (SZ) is a severe chronic mental illness that affects approximately 1% of the world population of both sexes (1, 2). Its symptoms are classified into positive symptoms (delusions, hallucinations general alterations, and cognitive and sensorial perceptions), negative symptoms (blunted affect, poor hygiene, depressed mood, and anhedonia), and cognitive impairment (3). Its causes and pathophysiology are not fully known yet.

This illness shows a complex multifactorial profile, involving several environmental, genetic, and psychosocial factors (2, 4, 5). The disease onset is frequently preceded by a subclinical symptoms phase (prodromal phase), associated with increased dopamine synthesis. However this association is observed only in patients who have developed mental illness later in life (5). The most accepted pathophysiological theory of SZ is the dopaminergic hypothesis (5, 6). This hypothesis is supported by the fact that all drugs used in SZ treatment have some action on the inhibition of dopaminergic receptors (7, 8). On the other hand, dopaminergic agonists, such as amphetamines, show an ability to precipitate psychotic symptoms (7).

The pathophysiology of SZ, according to this hypothesis, is related to a dopaminergic hypofunction on the prefrontal cortex (negative symptoms) and a secondary hyperfunction on the striated cortex (positive symptoms) (9). This theory has some inconsistencies because there is a gap of 2–4 weeks between the peak of D_2_ receptors blocked by the drug and the clinical response (10). Approximately one-third of patients show low or no improvement of symptoms with antipsychotic (AP) use, which motivates the search for new therapeutics targets (11).

Previously, it was believed that the central nervous system (CNS) was relatively protected from peripheric inflammatory processes due to the blood–brain barrier. However, this is not true because inflammatory processes increase the permeability of the blood–brain barrier and possibilities the leukocytes invasion in brain. Inflammatory processes have more importance in the acute phase, when invasions may occur to the CNS by macrophages and lymphocytes, while in the chronic phase, there a (12, 13). Taking this into account, a hypothesis is that the patient’s inflammatory profile may predict the response to clozapine and be useful in classifying the subtypes of SZ.

There is a growing number of recent studies that correlate inflammatory markers with psychiatric conditions such as mood disorders and suicidal behavior. Inflammatory processes in the hypothalamus–pituitary–adrenal (HPA) axis have been reported as involved in negative clinical outcomes regardless of the presence/absence of psychiatric conditions. Increased levels of stress are linked to HPA alterations and dysregulation of the serotoninergic system, including 5-HTTA receptors in individuals with mood disorders and suicidal attempts (14). Although well known, the origin and extent of this process are not well understood.

Moreover, subjects with abnormally elevated inflammatory markers may even be at higher risk of developing treatment resistance. Importantly, compared to non-suicidal subjects, higher mean concentrations of inflammatory mediators for treatment resistance have been found in both the periphery and brain of individuals who present with suicidal behaviors (15).

Considering that psychiatric practice today is still based on subjective diagnostic criteria, the discovery of biological markers as neutrophil-lymphocyte ratio (NLR) and immune inflammatory systemic index (SII), would play a great role in the credibility of diagnosis. It would aim to estimate the metabolic changes that can be caused by mental illness, clinical response, and tolerance to drugs such as clozapine. In this study, we evaluate the correlation between inflammatory markers [neutrophil, lymphocyte, platelets, IIS, NLR, and Monocyte-Linphocyte Ratio (MLR)], daily doses of clozapine, and the severity of psychopathology in SZ.

## Methods

This research work is a cross-sectional study with a control group (non-clozapine patients) and a trial group (clozapine patients). Patients were recruited from March 2022 to February 2023 from the Schizophrenia Program (PRODESQ) of a university hospital [*Clinical Hospital of Porto Alegre* (HCPA)], of a psychosocial care center [*Centro de Atendimento Psicossocial* (CAPS)] of the same hospital, and of a non-governmental mental health institution [*Associação Gaúcha dos Familiares de Pacientes com Esquizofrenia* (AGAFAPE)], all located in the city of Porto Alegre-RS, Brazil. The inclusion criteria were as follows:

Agreement to participate in the research study.

- A previous SZ diagnosis.
- Be between 18 and 70 years old.

For the trial group, besides these criteria, patients were required to be taking clozapine at a stable dose for at least 15 days.

The exclusion criteria were as follows:

- Patients with autoimmune disease.
- Patients with chronic inflammatory disease.
- Patients with severe cognitive impairment.

### Psychometric instruments

Within the scope of this study, we applied the Brief Psychiatric Rating Scale (BPRS). BPRS is a semi-structured 18-item instrument, utilizing a seven-point Likert scale, designed to quantify the intensity of psychopathological symptoms. This tool encompasses domains ranging from affective symptomatology, such as depression and anxiety, to psychotic manifestations such as hallucinations and delusions, with evaluations grounded in rigorous clinical observations and structured interviews. To the best of our knowledge, there is no study correlating inflammatory markers with BPRS. Thus, we considered a study to assess the correlation of inflammatory markers with the Positive and Negative Syndrome Scale (PANSS). This scale consists of 30 items categorized into three subscales: positive symptoms, negative symptoms, and general psychopathology. Each PANSS item is meticulously rated on a seven-point Likert scale, denoting the range from symptom absence to extreme severity.

### Sample size

We calculated the sample size to test whether the Pearson linear coefficient correlation between neutrophil count and PANSS score was different from 0. Calculations were made using the PSS Health tool (16). Considering a significance level of 5%, statistical power of 95%, and expected correlation of 0.223 according to Steiner et al. (17), we found a sample size of 255 patients. As this is a cross-sectional study, there was no loss to follow-up.

Despite the analyzed variables (leukocyte count and PANSS score) not being the same variables that were used in this research (IIS and BPRS), these scales (BPRS and PANSS) showed a strong correlation with each other (*p* = 0.96) (18). There appears to be a strong correlation between the other variables too once both are a leukocyte count being ISS a most sophisticated method only.

To the best of our knowledge, there are no studies that correlate clozapine use with SII and leukocyte count. Thus, we estimated a control group of 30 patients according to what was used by Hefner et al. (19).

### Ethical aspects

This study was approved by the HCPA ethical committee (project n° 2021/0016 CAAE n° 46901221500005327).

The patients who met the inclusion criteria were contacted by the research team and asked to participate in the study. Those who accepted were given further information about the study and were asked to sign the informed consent form. After this, patients were asked about clinical aspects that are found in patient records and then we applied the BPRS.

All data used were stored in the data bank and accessed by the software SPSS (*Statistical Package for the Social Sciences*) in virtual form. The data were stored on an institutional drive and was available only to the research team.

### Protocol

After agreeing to participate in the study, patients were asked about missing data on their patient records (hypertension, diabetes, time of disease, clozapine time of treatment, use of illegal drugs, hospitalizations, and suicide attempts). Then, BPRS was applied and the patients’ participation in the study ended.

### Statistical analysis

Statistical analysis was performed using the software SPSS (*Statistical Package for the Social Sciences*) version 27.0. The quantitative variables [age, BMI, time of SZ diagnosis, time of clozapine use, hospitalizations, systemic immune inflammation index (SII), and leukocyte ratio (NLR, MLR, and LPR)] were described as mean and standard deviation or median and interquartile range. The categorical variables (type of medication, drug use, BPRS score, and antibiotic or corticoid use) were described in absolute frequency and percentage.

The Student’s *t*-test was used to compare the mean between groups with and without medication use, such as clozapine and other antipsychotics. In the case of data asymmetry, the Mann–Whitney test was applied. Pearson correlation coefficient or Spearman correlation coefficient were applied to assess the correlation between inflammatory markers, BPRS scores, and clozapine dose. Spearman coefficient was used to assess the correlation between the BPRS score and each of the inflammatory markers to estimate a possible confusion bias between the clozapine daily dose and the BPRS score.

## Results

**Figure 1:**
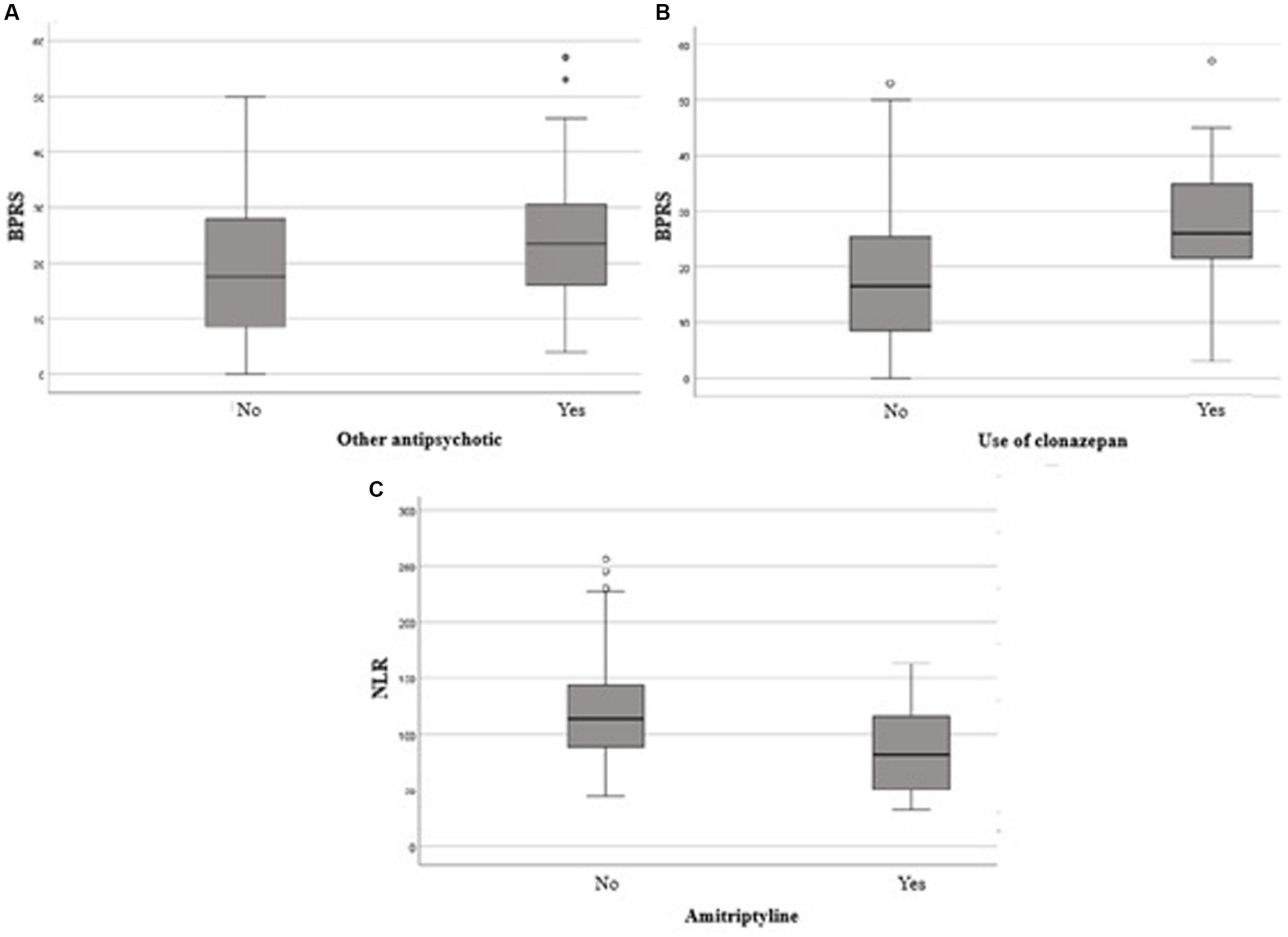
Comparison of total BPRS score between users and non-users of other antipsychotics **(A)**. Clonazepan **(B)** and comparison of NLR between users and non-users of amitriptyline. BPRS, Brief Psychiatric Rating Scaled; NLR,Neutrophil-lymphocyte ratio.

We initially assessed 116 patients’ records, with 12 being excluded because of diagnoses of overlapping brain injuries, chronic inflammatory processes, etc., as shown in Figure 1. After exclusions, 104 patients remained (78 men and 26 women).

The epidemiologic characteristics of the study’s sample (sex, age, literacy level, smoking, comorbidities, drug use, hospitalization, BMI, age of onset, and clozapine dosage) are described in Table 1.

**Table 1:** Demographic characteristics of the study’s sample.

Categorical variables	
Sex	
Male	78 (75%)
Female	26 (25%)
Literacy	
Incomplete Elementary school	19 (18.4%)
Complete Elementary school	33 (32.0%)
Incomplete High School	44 (42.7%)
Complete High School	7 (6.8%)
Smoking	19 (18.26%)
Comorbidity	81 (77.88%)
Diabetes	14 (13.46%)
Obesity	28 (26.92%)
Dyslipidaemia	17(16.34%)
Psychiatric hospitalization	83 (81.4%)
Drug use	
Clozapine	91 (87.5%)
Haloperidol	19 (18.3%)
Risperidone	11 (10.6%)
Other antipsychotics	40 (38.5%)
Clonazepam	31 (29.8%)
Amitriptyline	10 (9.6%)
Continuous variables	
Age (years)—mean ± SD.	46.4 ± 11.4
Duration (in years)—mean ± SD	24.3 ± 10.9
Monthly income (in basic salary^*^)—median (P25-P75)	2 (2–3)
BMI (kg/m^2^)—mean ± SD	27.0 ± 5.3
Dose of clozapine—mean ± SD	440.5 ± 163.0
Age of onset—mean ± SD	21.6 ± 6.2
Early-onset (<40 years)	100 (99.0%)
Late-onset (>40 years)	1 (1.0%)

^*^Basic salary in Brazil is about 264 US$. M, Male; F, Female.

The *Brief Psychiatric Rating Scale* (BPRS) was used to evaluate the severity of psychiatric symptoms. The highest score was registered for the items “somatic concern” (median: 1.5; interval: 0–3) and “anxiety” (median: 3; interval: 2–4). The BPRS total score median was 20 (interval:12–30).

The clozapine users showed a significantly higher neutrophil count (mean ± DP: 5.03 ± 2.07) in comparison to non-clozapine users (mean ± SD: 3.48 ± 1.27; *p* = 0.031) as shown in Table 2.

**Table 2:** Evaluation of inflammatory markers and BPRS according to the use or not of clozapine.

Variable	Clozapine users (*n* = 91)	Non-clozapine users (*n* = 13)	*p*
Inflammatory markers			
Neutrophils—mean ± SD	5.03 ± 2.07	3.48 ± 1.27	0.031^*^
Monocytes—mean ± SD	0.59 ± 0.21	0.59 ± 0.20	0.991
Lymphocytes—mean ± SD	2.06 ± 0.77	2.01 ± 0.56	0.828
Plaquets—mean ± SD	218.8 ± 64.8	193.1 ± 47.7	0.251
Neutrophil-lymphocyte ratio—median (P25–P75)	2.39 (1.53–3.48)	1.76 (1.12–2.60)	0.091
Monocyte-lymphocyte ratio—median (P25–P75)	0.29 (0.22–0.37)	0.32 (0.21–0.53)	0.820
Plaquets-lymphocyte ratio—median (P25–P75)	110.7 (84.8–143.6)	97.2 (65.0–146.8)	0.534
Immunoinflamatory systemic index—median (P25–P75)	538.9 (317.3–804.8)	333.4 (175.3–571.7)	0.051
BPRS total score median (P25–P75)	20 (12–30)	19 (16–27)	0.655

Although there were no significant differences related to other parameters, clozapine users seem to present a tendency to have higher inflammatory markers, except for monocytes, which was equal, and for MLR, which showed a lower count in clozapine users. These results are presented in Table 3.

**Table 3:** Evaluation of the immuno-inflammatory systemic index between clozapine users and non-clozapine users controlling for comorbidities.

Variable	Clozapine users	Non-clozapine users	*p*
Without any comorbidity	21	4	
IIS	570.5 (352.6–807.5)	247.3 (352.6–807.5)	0.0189^*^
NLR	2.720 (1.811–3.315)	1.6210 (1.2747–1.8832)	0.0700
Without SAH	80	9	
IIS	542,9	333,4	0.0419^*^
NLR	2.4964 (1.62–3.48)	1.7633 (1.25–2.24)	0.078
Without dyslipidemia	73	9	
IIS	569.2 (345.5–843.4)	333.4 (197.5–5,383)	0.021^*^
NLR	2.6250 (1.71–3.49)	1.7633 (1.25–2.24)	0.057

The correlation between inflammatory markers and daily dose of clozapine (DDC) was evaluated, but no statistical significance was found for any evaluated parameters (Table 4).

**Table 4:** Association between inflammatory markers and daily dose of clozapine.

Variables	DDC × MI	*p*
Inflammatory markers		
Neutrophils^*^	-0.017	0.876
Monocytes^*^	0.089	0.408
Lymphocytes^*^	0.003	0.980
Platelets^*^	-0.098	0.363
Neutrophil-lymphocyte ratio^**^	0.007	0.947
Monocyte-lymphocyte ratio^**^	0.036	0.740
Platelets-lymphocyte ratio^**^	-0.058	0.593
Immune inflammatory systemic index^**^	-0.034	0.753
BPRS total score^**^	0.074	0.492

^*^Pearson’s correlation coefficient, ^**^Spearman’s correlation coefficient. DDC, Daily dose of clozapine. IM, Inflammatory markers.

Furthermore, no correlation was found between inflammatory markers and the severity of symptoms.

Patients using APs other than clozapine showed significantly higher BPRS scores than those who did not use any AP medication (*p* = 0,036). Furthermore, the use of clonazepam and amitriptyline was associated with significantly higher BPRS scores (*p* < 0,001 e *p* = 0,047, respectively).

## Discussion

Treatment with clozapine showed a significantly higher neutrophil count among clozapine users than non-clozapine users (*p* = 0,031). These findings are in agreement with an experimental study in animal models. In this study, an increase in neutrophils and a decrease in lymphocyte counts were observed a few hours after a single dose of clozapine (20).

Our study also showed that patients who use amitriptyline and/or other antipsychotic drugs have higher BPRS scores than patients who do not use these drugs. This finding is probably due to the ultra-refractory profile of these patients, indicating that the symptomatic manifestation remains resistant to treatment, including with clozapine use. The majority of patients included in this study were men (75%). Despite epidemiological studies showing a higher incidence of schizophrenia in male individuals, and the male/female ratio being 1:4 without significant difference in prevalence, these conclusions could not be verified because this is a cross-sectional study (21). A shown hypothesis in a related study suggests that the discrepancy between incidence and prevalence of schizophrenia could be due to differences in treatment adherence between the sexes and higher suicide rates associated with the male sex (22). This factor can affect the prevalence of schizophrenia up to five times, as shown in a study that assigned these variations to applied methodology, different risk factors, and the predominance of some regional genetic profiles (23).

It was also observed that schizophrenia tends to manifest a bit earlier in men than in women and that refusal to participate in treatment, which tends to be higher in men than women, leads to a worse prognosis. Despite women (median of 19 years) having a lower age of onset than men (median of 20 years), this difference was not significant (*p* = 0,4,844). The lack of statistical significance could be explained by the small sample size, as the sample does not show a normal distribution for age of onset (Shapiro–Wilk: W = 0.93155, *p* < 0,005). In addition, the BPRS score also did not show a significant difference between men and women (*p* = 0,172).

These findings suggest a need to consider sample asymmetry and a possible selection bias because patients with severe cognitive impairment were excluded from the research due to the impossibility of applying the BPRS, as well as refusal to participate in a significant proportion of patients, which probably indicates a worse psychopathological condition.

When classified in early-onset (less than 40 years) and late-onset (more than 40 years) (24), it was observed that almost all patients had early onset (less than 40 years), with only one patient having a late onset of the disease. This fact is according to several studies that show that schizophrenia onset is more frequent in early adulthood and rare in old age. These studies also suggest that schizophrenia could have distinct courses depending on the age of onset, supporting the hypothesis that late-onset schizophrenia could be considered a subtype of the disease (19).

### Smoking

Among SZ patients 18,26% were smokers. Substance use, such as tobacco and alcohol, is often used as a way of coping with anxiety and stress both in the average population and in psychiatric patients (25). However, patients with SZ often smoke to relieve drug adverse reactions, such as those known as “Parkinsonian Syndrome.” These symptoms are mainly caused by first-generation AP drugs, dopaminergic antagonists that cause a dopaminergic depletion in motor cortical, which triggers extrapyramidal symptoms (26).

Smoking is common in patients with mental disorders including patients with SZ. In this study, 18,23% of patients were smokers. It is known that the prevalence of smoking among patients with SZ is higher than in the average population and that these patients could have up to a five times greater risk because of their smoking habits (27). Patients with SZ also show a lower ratio (approximately two times lower) for smoking cessation than the average population; this difficulty seems to be related to cognitive impairment in SZ (28).

Smoking is also one of the main associated factors in the mortality increase in severe mental disorders, such as schizophrenia (29). Smoking has a great impact on the pharmacotherapy of patients with schizophrenia since tobacco interacts with most AP drugs. Tobacco increases AP metabolism by induction of the cytochrome P450 1A2 isoform. In addition, it decreases serum levels of first- and second-generation AP drugs, such as haloperidol, chlorpromazine, olanzapine, and clozapine (28).

Tobacco has a known inflammatory effect, mainly in the lung, but also causes tissue damage in other organs. Smoke exposure leads to oxidative/anti-oxidative imbalance, leading to oxidative stress and increased pro-inflammatory cytokine expression (30).

### Comorbidities

Comorbidities are common in SZ and are associated with an increase in mortality in patients with SZ. In this study, obesity was the most frequent comorbidity (26.9%). Obesity and mental disorders consist in a significant parcel of global disease, according to the *National Obesity Observatory* (31).

The World Health Organization estimates that 40% of obese adults have a well-established relationship between their medical conditions and obesity. However, the association between obesity and some psychiatric diseases specifically, such as SZ, is still poorly studied (32). An association between Body Mass Index (BMI) and the prevalence of mental illness shows that individuals with a BMI greater than 30 have a prevalence rate greater than 56.7 for all mental illnesses; in comparison, the prevalence rate for individuals with a BMI of less than 19 is 44.8% (33).

One of the mechanisms through which SZ leads to cardiometabolic risk factors, such as obesity, systemic arterial hypertension (SAH), and dyslipidemia, is through inflammatory pathways. This agrees with the fact that psychiatric diseases such as SZ are associated with several increased inflammatory markers (34).

These findings agree with other studies, however, it is difficult to know whether obesity is related in fact as a metabolic disorder to SZ or if it is related to the lifestyle of patients, who in general tend to be more sedentary (35). Another important factor to be considered is the weight provided by several AP drugs. It is well-known that AP drugs, mainly second-generation ones, lead to the occurrence of metabolic disorders. Some studies show an association between these AP drugs and cardiovascular effects, resistance to insulin, obesity, and dyslipidemia (36–38).

### Clozapine and inflammatory markers

Patients who used clozapine showed a greater neutrophil count than those who did not use the drug (*p* = 0,031). Although it seems counterintuitive, some studies have already associated clozapine and inflammatory effects such as myocarditis and fever (39–41).

Clozapine may lead to an adverse reaction, such as leukopenia and agranulocytosis that causes a decrease in leukocyte count. These adverse reactions occur in 1–2% of patients (42). However, the mechanism by which this occurs is still not well-understood in humans. A study that evaluated myocarditis induced by clozapine in mice showed an increase in catecholamines in animals treated with clozapine, thus proposing it as a possible mechanism of induction of myocarditis. However, there are no studies that evaluate this hypothesis in humans (43).

A systematic review evaluated the outcomes related to clozapine immunomodulatory effects (44). There is a relationship between clozapine effects with interleukin production and autoimmune disease. However, there is conflict in this aspect as some studies have shown no difference. Regarding adaptive immunity involving lymphocytes, several discrepancies were observed. Three studies showed an increase in IFNG production (45–47), while one study showed a decrease (48). Clozapine also shows an influence on pro-inflammatory cytokines. A study observed an inhibitory effect of a clozapine metabolite (norlcozapine) on tumor necrosis factor (TNF), but a stimulant effect of clozapine on IL 17 (49). Effects on IL 2 were also observed and three studies showed inhibition of clozapine on this interleukin (47–49); however, another study showed the opposite (46).

Studies *in vivo* show a different action of clozapine in the course of the disease, with a significant increase of TNF and the receptors sTNRF1 e sTNRF2 during the first 6 weeks of clozapine treatment. However, after 10 weeks of treatment, there was a normalization (50, 51).

The inflammatory ratio NLR tends to be higher among those who do not use clozapine. However, when controlled for comorbidity in general, SAH, and dyslipidemia, NLR was significantly higher in clozapine users than in non-clozapine users, as shown in Table 3.

### Study methodological and contextual limitations

The overrepresentation (75%) of male subjects may introduce a selection bias, limiting the extrapolation of findings to the broader schizophrenic spectrum. The cross-sectional approach employed restricts the potential for causal inferences. Furthermore, the geographical delimitation of the sample does not capture the inherent clinical and epidemiological diversity of schizophrenia across different regions. The statistical power, compromised by the sample size, is underscored by the lack of significance in the analysis of disease onset age between the sexes.

## Conclusion

This study has shown an association between clozapine use and neutrophil increase, such as an increase of SII and NLR, considering control of SAH and dyslipidemia. These results agree with previous studies that showed an inflammatory response due to clozapine administration. Furthermore, amitriptyline shows a decrease in SII and NLR, corroborating research that suggested an anti-inflammatory effect of tricyclic antidepressants.

An expressive neutrophil increase was observed and a trend in the ratio may indicate significance in multivariate analysis in subgroups of patients. Therefore, such is the importance of these aspects to future research, which should aim to better understand the role of inflammatory markers in SZ and their treatment.

The increase in BPRS scores in patients in treatment with other AP drugs and/or clonazepam may indicate the severity of symptoms in these patients. However, according to preview studies, no correlation was observed between inflammatory markers and BPRS score. Countering the lack of correlation between inflammatory markers and the clinical state of patients with SZ, our results suggest that such markers are relevant in the preview of clinical responses to clozapine. It becomes evident, however, that there is a need to expand the sample and stratify the patients according to different comorbidities in future studies. Despite this, the inflammatory profile has been shown to be an important factor that must be considered by clinicians in prescribing clozapine. Measuring the serum dosage of clozapine and cohort studies are needed to confirm the findings and assess the causality between inflammatory markers, disease course, and response to clozapine.

## Data availability statement

The original contributions presented in the study are included in the article/supplementary material, further inquiries can be directed to the corresponding author.

## Ethics statement

The studies involving humans were approved by Committee of Ethics in Research at Hospital de Clínicas de Porto Alegre. OHRP/EUA (Office for Human Research Protections): IORG0000588. IRB 00000921 Federalwide Assurance (FWA) HHS (U.S. Department of Health & Human Services) Protection of Human Subjects: FWA00002409. The studies were conducted in accordance with the local legislation and institutional requirements. The participants provided their written informed consent to participate in this study. Written informed consent was obtained from the individual(s) for the publication of any potentially identifiable images or data included in this article.

## Author contributions

VHSC: Conceptualization, Investigation, Methodology, Project administration, Writing – original draft, Writing – review & editing. ADT: Conceptualization, Data curation, Investigation, Visualization, Writing – review & editing. APA: Methodology, Visualization, Writing – review & editing. RM: Visualization, Writing – review & editing. PSB-d-A: Funding acquisition, Investigation, Methodology, Project administration, Resources, Supervision, Visualization, Writing – review & editing.
